# Enhanced ferroelectric switching speed of Si-doped HfO_2_ thin film tailored by oxygen deficiency

**DOI:** 10.1038/s41598-021-85773-7

**Published:** 2021-03-18

**Authors:** Kyoungjun Lee, Kunwoo Park, Hyun-Jae Lee, Myeong Seop Song, Kyu Cheol Lee, Jin Namkung, Jun Hee Lee, Jungwon Park, Seung Chul Chae

**Affiliations:** 1 Department of Physics Education, Seoul National University, Seoul, 08826 Korea; 2 School of Chemical and Biological Engineering, Institute of Chemical Process, Seoul National University, Seoul, 08826 Korea; 3 Center for Nanoparticle Research, Institute for Basic Science (IBS), Seoul, 08826 Korea; 4 School of Energy and Chemical Engineering, Ulsan National Institute of Science and Technology (UNIST), Ulsan, 44919 Korea

**Keywords:** Electronic properties and materials, Ferroelectrics and multiferroics

## Abstract

Investigations concerning oxygen deficiency will increase our understanding of those factors that govern the overall material properties. Various studies have examined the relationship between oxygen deficiency and the phase transformation from a nonpolar phase to a polar phase in HfO_2_ thin films. However, there are few reports on the effects of oxygen deficiencies on the switching dynamics of the ferroelectric phase itself. Herein, we report the oxygen- deficiency induced enhancement of ferroelectric switching properties of Si-doped HfO_2_ thin films. By controlling the annealing conditions, we controlled the oxygen deficiency concentration in the ferroelectric orthorhombic HfO_2_ phase. Rapid high-temperature (800 °C) annealing of the HfO_2_ film accelerated the characteristic switching speed compared to low-temperature (600 °C) annealing. Scanning transmission electron microscopy and electron energy-loss spectroscopy (EELS) revealed that thermal annealing increased oxygen deficiencies, and first-principles calculations demonstrated a reduction of the energy barrier of the polarization flip with increased oxygen deficiency. A Monte Carlo simulation for the variation in the energy barrier of the polarization flipping confirmed the increase of characteristic switching speed.

## Introduction

Defects, such as oxygen deficiencies, are present in all material systems and are generally considered detrimental to performance. Extensive attention has been paid to the role of defects and their reduction^1^. However, defects do not always suppress functionality and by controlling them diverse functionality can be achieved, such as incipient magnetism in two-dimensional materials^2^, active sites for water dissociation^3^, and the conventional manipulation of semiconductor conductivity. Considering the double-sidedness of defects, a profound understanding of them is required to control material functionality^4^. In particular, due to the importance of the structural composition of conventional perovskite oxides, numerous investigations have focused on the effects of defects on ferroelectric properties. Usually, defects in ferroelectric materials are considered to be the origin of the degradation of ferroelectric properties, which include ferroelectric domain pinning, fatigue, and imprint^5^. However, the control of oxygen stoichiometry has also been considered a promising way to manipulate the functionality of ferroelectric oxides, such as incipient ferroelectricity^6^, stabilization of intermediate polarization states^7^, and defect-mediated polarization switching^8,9^.

The unprecedented discovery of non-centrosymmetric inversion symmetry-breaking and spontaneous polarization in HfO_2_ thin films has shed renewed light on the feasibility of ferroelectric logic and memory device applications^10–12^. Ferroelectric HfO_2_ is considered an alternative to ferroelectric perovskites because of its compatibility with current complementary metal–oxide–semiconductor (CMOS) technologies and high scalability. A large remnant polarization of 10–40 μC/cm^2^ can be obtained for HfO_2_ films with the orthorhombic Pca2_1_ phase^13,14^. In addition, diverse electric properties with structural changes can be realized via dopant control and electric field cycling^15,16^. Integration of HfO_2_ films with the CMOS process will aid the development of next-generation non-volatile logic and memory applications.

The functionality of spontaneous polarization of HfO_2_ film has been characterized at the fundamental scientific and device application levels. Robust scalability with a sufficiently large coercive field has enabled applications requiring a wide memory window^17–19^. In addition, the robust scalability of ferroelectric polarization has enabled device fabrication at the sub-10 nm scale. Structural analyses of an ultrathin HfO_2_ film revealed that its ferroelectricity was due to the non-centrosymmetric orthorhombic Pca2_1_ phase caused by strain in the film^20^. Recently, ferroelectricity in a literally 1-nm thick ultrathin HfO_2_ film was observed with enhanced polar distortion using piezoresponse microscopy and second harmonic generation measurements^21^. Furthermore, the proximity effects of a nonmetallic interface in the form of a field-effect transistor with insulator and semiconductor contacts were investigated with respect to the stability of ferroelectric polarization^22,23^. Robust subloop polarization stability of the ferroelectric HfO_2_ film has also been reported for deterministic control of memory states for analog devices^24,25^. The small critical volume for ferroelectric nucleation can be stabilized by weak interactions with neighboring ferroelectric dipole moments, thereby enabling robust subloop polarization stabilization^26^. The extremely weak interaction between ferroelectric dipoles was attributed to the flat ferroelectric phonon, which is a unique character of HfO_2_-based ferroelectricity^27^. These causalities in HfO_2_ itself and the consequent emergence of ferroelectricity have been pursued to understand the underlying mechanism of ferroelectricity more precisely.

Considering the importance of defects to ferroelectric functionality, the effects of phase transformation in ferroelectric HfO_2_ have been extensively studied. For example, a correlation between orthorhombic phase formation and oxygen deficiency was proposed during an investigation of the enhancement of remnant polarization under electric field cycling: The migration and redistribution of oxygen deficiencies in the middle of the film were coincident with the formation of the orthorhombic phase, which enhanced polarization^28^. However, the role of oxygen deficiency on the dynamic properties, e.g., ferroelectric polarization switching, has rarely been studied. Further investigation concerning oxygen deficiency will increase our understanding of the key factors governing the overall ferroelectric properties. In this study, we enhanced ferroelectric switching properties in terms of switching speed and homogeneity by controlling oxygen deficiency.

## Results

**Figure 1:**
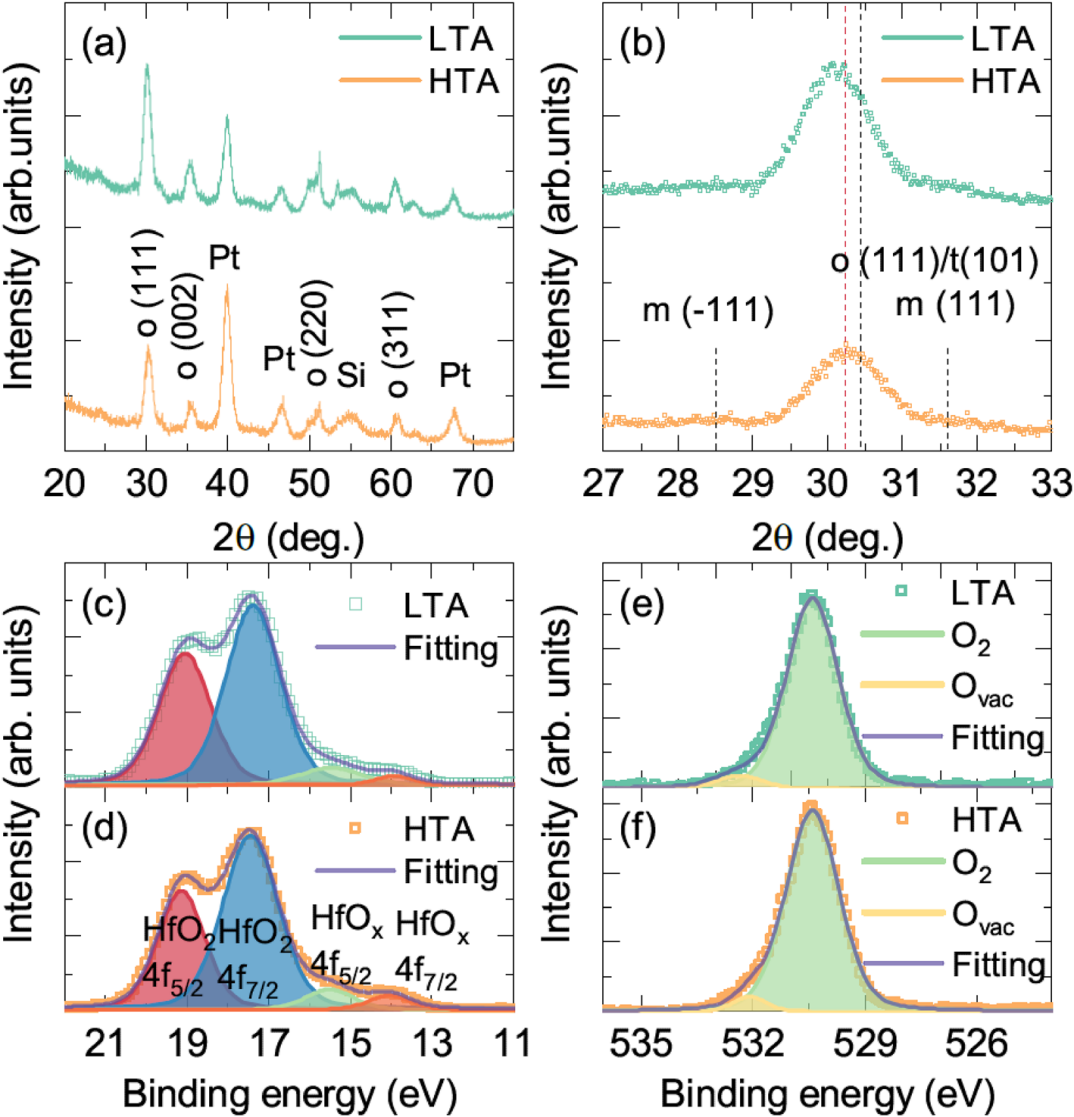
Structural and chemical analyses of HfO_2_ films. (**a**) Grazing incidence X-ray diffraction (GIXRD) results after annealing at 600 °C for 20 s (LTA) and 800 °C for 1 s (HTA). (**b**) Enlarged GIXRD results near the orthorhombic (111) peaks. X-ray photoemission spectroscopy results near the Hf 4f peak for (**c**) LTA and (**d**) HTA films and O 1*s* peak for (**e**) LTA and (**f**) HTA films.

Polymorphism of polycrystalline HfO_2_ thin films was suppressed during the atomic layer deposition process followed by post-annealing (see the Experimental Section). Figure 1a shows the grazing incident X-ray diffraction patterns of low-temperature annealing (LTA) and high-temperature annealing (HTA) films. Both films contained the orthorhombic/tetragonal phase with negligible monoclinic phase (Fig. 1a). However, a structural distortion was observed for the different annealing temperatures (Fig. 1b). Enlarged X-ray diffractograms revealed a shift of the diffraction peak of the orthorhombic (111)/tetragonal (101) phase near 2*θ* ~ 30°. The peak shift could be attributed to the phase transition from orthorhombic to tetragonal^13^ and/or the smaller lattice parameters of the orthorhombic phase caused by more oxygen deficiencies in the fluorite structure^29,30^. The strong competition between electrostatic interaction and the steric effect resulted in the volume contraction of the non-stoichiometric fluorite structure. Structural instability of a polymorphic HfO_2_ film can modulate the ferroelectric properties. Notably, for Si-doped HfO_2_ films, the ferroelectric remnant polarization increased with decreasing orthorhombic (111)/tetragonal (101) lattice parameters^31^. Considering the different peak positions for the LTA and HTA films, distinct ferroelectric properties of both films were expected.

The mediation of oxygen deficiency in the Si-doped HfO_2_ films was coincident with the change in annealing condition. To confirm the effect of the annealing process on the oxygen deficiency concentration, X-ray photoemission spectroscopy (XPS) was used to analyze the chemical states of HfO_2_ after Ar^+^ ion etching. The XPS results near the Hf 4f, O 1*s* peaks of the LTA and HTA films were used to compare the oxygen deficiencies concentration (Fig. 1c–f). Figure 1c,d shows the Hf 4f core-level spectra near 17 eV. The spectra of both LTA and HTA films can be considered to be the complex of doublet peaks of stoichiometric HfO_2_ and off-stoichiometric HfO_x_ due to the oxygen vacancies^32,33^. For the quantitative analysis of oxygen vacancies, the Hf 4f spectra were carefully deconvoluted through 4 components in terms of HfO_2_ ${4f}_{5/2}$, ${4f}_{7/2}$ and HfO_x_ ${4f}_{5/2}$, ${4f}_{7/2}$. The HfO_x_ concentration was estimated to 7.8% and 10.5% from the peak intensity ratios (I_Hf_) of LTA and HTA films, respectively. O 1* s* peak near 530 eV was accompanied by satellite peaks as shown in Fig. 1e,f. These satellite peaks corresponded to hydroxyl groups^34,35^. The estimated peak intensity(I_O_) ratios between the low-energy satellite shoulder peak and total oxygen peak intensity were 4.4% and 5.6% for the LTA and HTA film, respectively. Considering that an oxygen vacancy releases 2 electrons and 2 molecular units in a unit cell^36^, the oxygen vacancy concentration can be estimated as 1/8 × I_Hf_ and 1/4 × I_O_. Therefore, the oxygen vacancy concentration of LTA and HTA films can be estimated to be 1.1% and 1.4% from Hf spectra and 1.0% and 1.3% from O spectra, respectively. These XPS results demonstrated that off-stoichiometry was enhanced in the HTA film.

**Figure 2:**
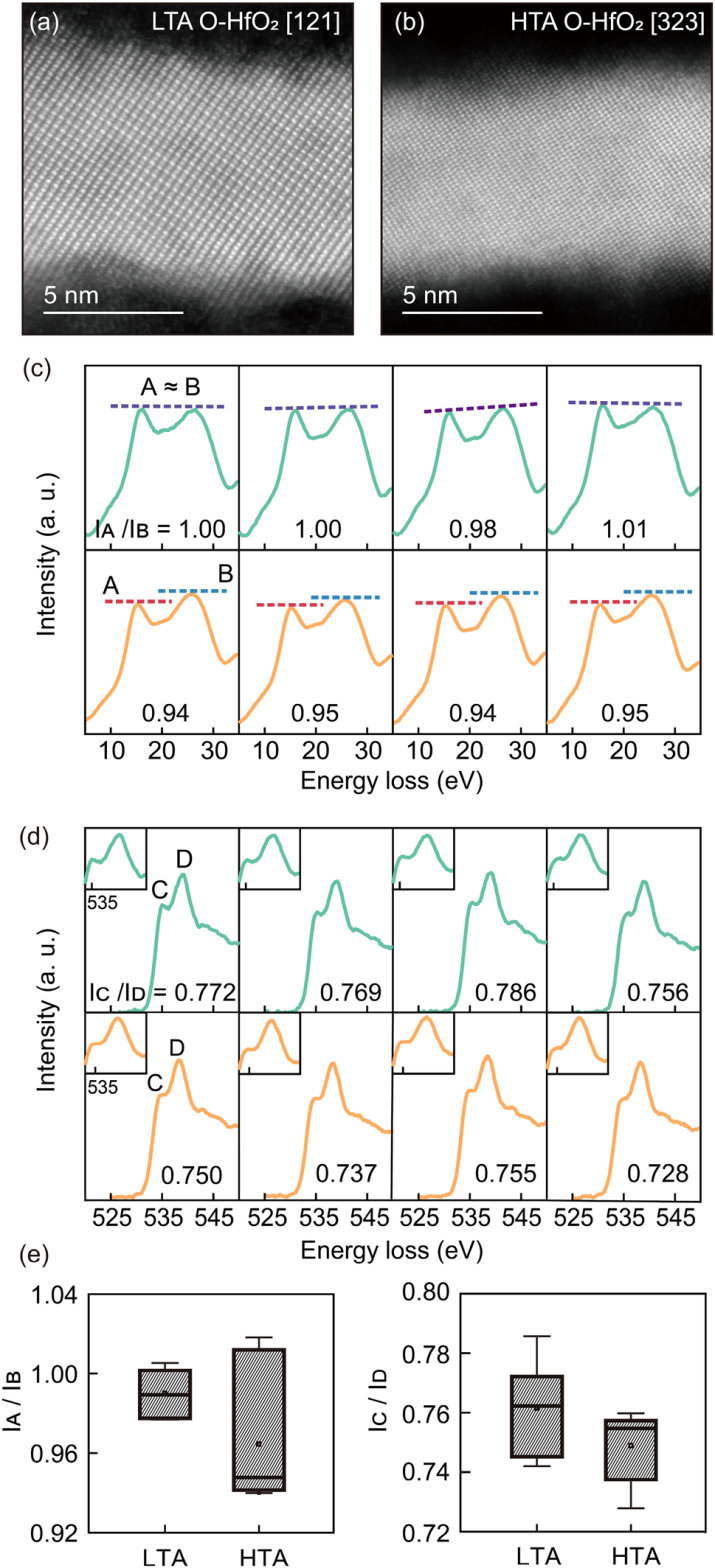
Cross-sectional images of orthorhombic HfO_2_ using the high-resolution scanning transmission electron microscopy (**a**) along zone axis [121] of the LTA film and (**b**) along zone axis [323] of the HTA film. (**c**) Valence electron energy-loss spectroscopy results and (**d**) O *K* edges showing different intensity ratio of peaks in the LTA (green) and HTA (orange) films. Insets show the double-peaked shape of the O *K* edges. (**e**) Box plots of the intensity ratios of peak A, B (left), and C, D (right).

Microscopic structural and chemical analyses using scanning transmission electron microscopy (STEM) manifested that high-temperature annealing of the HfO_2_ film introduced more oxygen deficiencies with the negligible phase transformation in the ferroelectric orthorhombic phase. Local chemical analyses of the ferroelectric phase were carried out using conventional high-resolution STEM (HRSTEM) and electron energy-loss spectroscopy (EELS). Figure 2a,b presents HRSTEM images of the LTA and HTA films. The zone axes and crystallographic structure were characterized by their fast Fourier transform (FFT) patterns (see supplementary Sect. 1). Both LTA and HTA films exhibited an orthorhombic Pca2_1_ phase with a negligible phase difference between the films. Also, the grain size estimation exhibited negligible differences between LTA and HTA films (see supplementary Sect. 2).

Differences in electronic structures were revealed by valence EELS (VEELS) and the O *K* edges. The local chemical analyses of the LTA and HTA films by EELS are shown in Fig. 2c,d. The VEELS spectra of both HfO_2_ films displayed two peaks, A (~ 16 eV) and B (~ 24 eV), which correspond to the plasmon excitation peaks of bulk HfO_2_ (see supplementary Sect. 3)^37^. Although the intensities of peaks A and B were similar in the LTA film, peak B was more intense than peak A in the HTA film. The lower intensity of peak A of the HTA film was attributed to weakened plasmon excitation caused by a higher oxygen deficiency concentration^38^. Likewise, the O *K* edges exhibited distinct double peaks (C and D) due to metal e_g_–t_2g_ splitting of the Hf 5*d* orbitals (Fig. 2d)^39^. Double-peaks at O *K* edges have been reported for HfO_2_ with various crystal structures^37–39^. While pronounced splitting of O *K* edges was observable in the LTA film, the HTA film exhibited the diminish of peak splitting. The diminish of peak splitting of O *K* edges was due to the higher oxygen deficiency concentration in the HTA film^38^. Fig. 2e shows the I_A_/I_B_ and I_C_/I_D_ ratios collected from various regions of both films. The average results also indicate that the HTA film had a higher oxygen deficiency concentration than the LTA film. The structural and chemical analyses indicated that the oxygen deficiency concentration in Si-HfO_2_ could be controlled with negligible phase transformation via short duration, high-temperature annealing.

**Figure 3:**
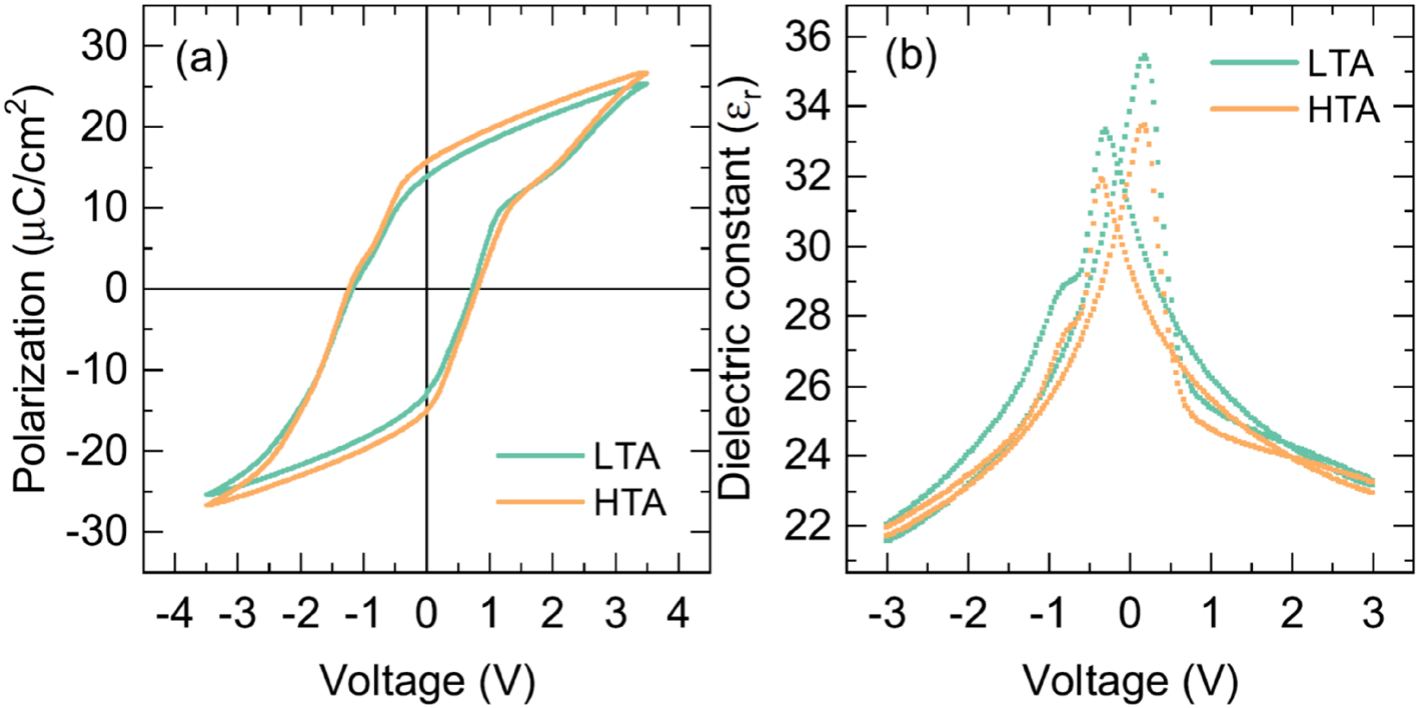
Macroscopic electrical properties of the HfO_2_ films. (**a**) Polarization–voltage hysteresis of the LTA (green line) and HTA (orange line) films. (**b**) Capacitance–voltage measurement of the LTA (green squares) and HTA (orange squares) films.

The degree of oxygen deficiency affected the ferroelectric behavior of the HfO_2_ thin films. Figure 3a shows the ferroelectric polarization hysteresis loops of the LTA and HTA films. Both films exhibited pinched hysteresis with asymmetric coercive voltage. During the HfO_2_ film growth, more defects such as oxygen vacancy, trap sites, and defect dipoles are generated at the bottom interface due to the high temperature growth process^40^. The asymmetric defects at the interfaces during the annealing process can induce asymmetric coercive voltages as well as pinched hysteresis (see supplementary Sect. 4)^7^. The remnant polarization, positive coercive voltage (V_c+_), and negative coercive voltage (V_c–_) of the LTA film were 13.3 μC/cm^2^, 0.73 V, and − 1.17 V, respectively, whereas those of the HTA film were 15.3 μC/cm^2^, 0.80 V, and − 1.23 V. Thus, the values were about 15%, 9%, and 5% higher for the HTA films. The polarization hysteresis loop for the oxygen deficiency-rich HfO_2_ film indicated robust ferroelectricity with enhanced remnant polarization and coercive voltages.

Counterintuitively, the oxygen deficiency-rich HfO_2_ film exhibited more homogenous ferroelectric switching^8^. Fig. 3b shows the dielectric constant as a function of voltage. Typical butterfly-like capacitance–voltage loops, which are characteristic of ferroelectric materials, were observed for both films. Comparing the similar dielectric constants at the high voltage where the capacitance contribution by ferroelectric switching can be negligible, the different annealing conditions of the two films had little effect on the polymorphic mixture of orthorhombic and tetragonal phases. Considering the different dielectric constants of monoclinic, orthorhombic, and tetragonal HfO_2_, it can be assumed that negligible phase transformation occurred, in agreement with the results shown in Fig. 2a,b^41^. On the other hand, the peak height near the coercive voltage was smaller for the HTA films than the LTA ones. With respect to the lower dielectric constants along the polarization direction in conventional ferroelectric materials^42–44^, the reduced dielectric constant peak in the oxygen deficiency-rich film can be considered uniformly poled polarization along the field direction.

**Figure 4:**
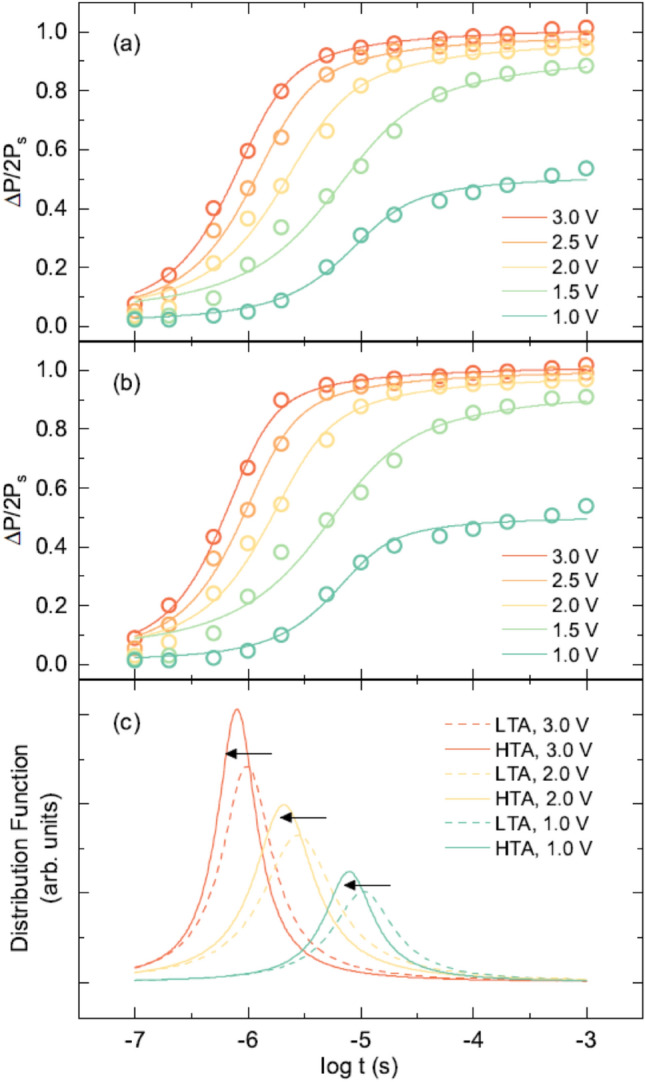
Ferroelectric switching dynamics of the HfO_2_ films. Time and voltage dependence of the ΔP(t)/2P_s_ values of the (**a**) LTA and (**b**) HTA films. The solid lines represent the fitting results obtained using the NLS model with a Lorentzian distribution of the characteristic switching time. (**c**) Fitting results of the Lorentzian distribution of the characteristic switching time for LTA (dashed lines) and HTA (solid lines) films.

In addition, the faster ferroelectric characteristic switching with less resistance to the external electrical stimulus indicated enhanced ferroelectric robustness in the HTA film. Considering the distribution of characteristic switching time due to the presence of dipole defects and the interaction of polarization, the nucleation limited switching (NLS) model with a Lorentzian distribution of characteristic switching time was used to evaluate the characteristic switching time and the homogeneity of distribution function (see supplementary Sect. 5)^45^. To estimate the ferroelectric characteristic switching times of the Si-doped HfO_2_ films, we measured ferroelectric switching dynamics in terms of voltage height and width. Figure 4a,b show the time and voltage dependence of ΔP(t)/2P_s_ values in the LTA and HTA films, respectively. The HTA film with high oxygen deficiency exhibited a counterintuitively sharper distribution with faster characteristic switching than the LTA film as shown in Fig. 4c. Intuitively, enhanced film homogeneity with respect to defects and/or the ferroelectric phase results in a sharper distribution with slower ferroelectric characteristic switching time^46^.

**Figure 5:**
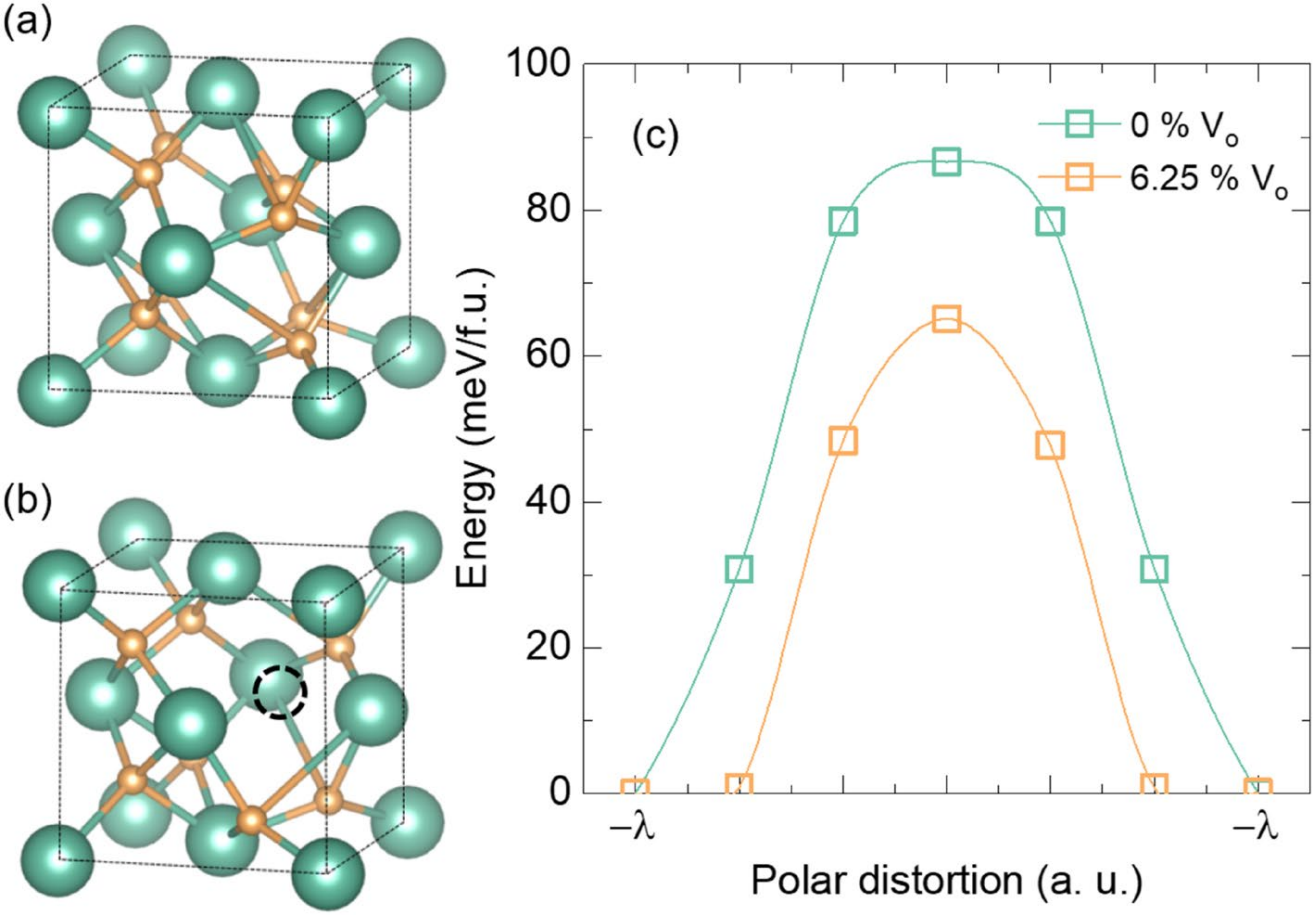
Schematic diagram of orthorhombic HfO_2_ (**a**) without oxygen deficiency and (**b**) with 6.25% oxygen deficiency. (**c**) First-principles calculations of the energy landscape during single ferroelectric dipole flipping for 0% and 6.25% oxygen deficiency concentrations.

Theoretical calculations indicated that the enhanced oxygen deficiency concentration reduced the energy barrier for the ferroelectric dipole flipping. First-principle calculations estimated the effects of oxygen deficiency concentration on the ferroelectric switching. Figure 5a,b show schematics of the atomic configurations of downward polarization in ferroelectric HfO_2_ without oxygen deficiency and with 6.25% oxygen deficiency, respectively. The oxygen deficiency-rich orthorhombic phase exhibited smaller lattice parameters and larger remnant polarization than the deficiency-free orthorhombic phase (see supplementary Sect. 6). Notably, the experimental results revealed a reduced (111) lattice parameter with increasing oxygen deficiency (Fig. 1d). Figure 5c illustrates the energy barrier for ferroelectric dipole flipping for different concentrations of oxygen deficiency. The calculated activation energy was 86.6 and 65.1 meV/f.u. for orthorhombic HfO_2_ without oxygen deficiencies and with 6.25% oxygen deficiencies, respectively. Note that due to the complex nature of ferroelectric switching^47,48^, the coercive field can be larger with small activation energy in certain circumstances which is not the inconsistency with experimental results^9^. Oxygen deficiency-rich HfO_2_ exhibited the smaller energy barrier of dipole flip than oxygen deficiency-free HfO_2_.

**Figure 6:**
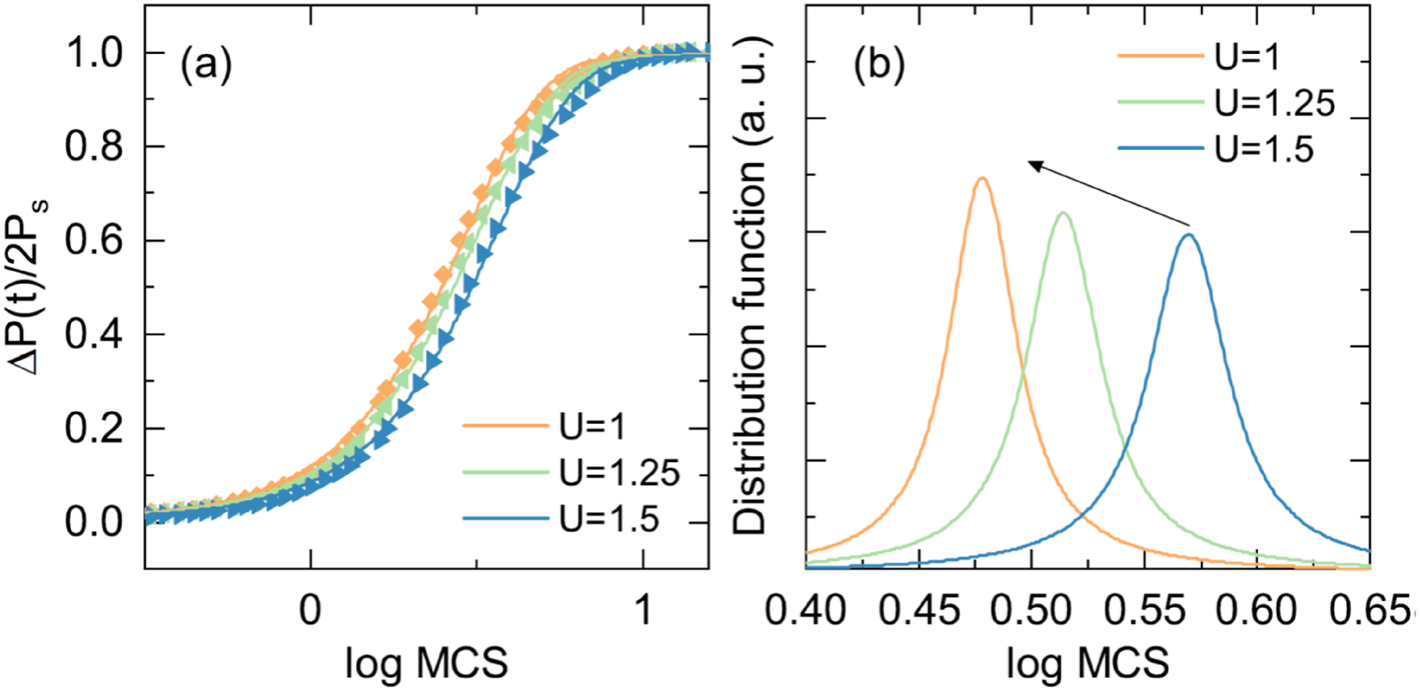
Monte Carlo simulations of the ferroelectric switching dynamics for different activation energy barrier heights. (**a**) Polarization reversal behavior and fitting line for different activation energy barriers. (**b**) Fitted Lorentzian distribution function for different activation energy barriers.

The causality between uniform switching and oxygen deficiency was investigated via Monte Carlo simulations in terms of the activation energy for ferroelectric switching. We conducted the Monte Carlo simulations to elucidate the effects of the activation energy of ferroelectric dipole flipping on the ferroelectric switching dynamics. The simulation was conducted for long-range dipole–dipole interactions^49^ with a certain energy barrier value^50^ using the Hamiltonian and switching probability as follows:

1 $$H = \sum\limits_{j} {\frac{{P_{i} \cdot P_{j} - 3\left( {P_{i} \cdot n} \right)\left( {P_{j} \cdot n} \right)}}{{r^{3} }} - P_{i} } \cdot E_{ext}$$

2 $$p_{i} = \left\{ {\begin{array}{ll} {\exp \left( { - \frac{\Delta H + U}{{kT}}} \right)} & {{\text{for}}\;\Delta H > 0} \\ {\exp \left( { - \frac{U}{kT}} \right)} & {{\text{for}}\;\Delta H < 0} \\ \end{array} } \right.$$

where *p*_*i*_, *P*_*i*_, *r*, *n*, *E*_*ext*_, and *U* represent the probability, ferroelectric dipole moment at lattice site *i*, distance between dipoles, unit vector connecting dipoles, external electric field, and energy barrier, respectively (see supplementary Sect. 7). Figure 6a shows the Monte Carlo step (MCS) dependence of ferroelectric polarization switching for different activation energy barriers. The Lorentzian distributions were sharpened with decreasing energy barrier as shown in Fig. 6b. The results reflect the relationship between activation energy and homogeneous ferroelectric switching. More uniform and faster switching was achieved in the case of lower activation energy which corresponds to the oxygen deficiency-rich ferroelectric HfO_2_.

The faster ferroelectric characteristic switching was attributed to increased oxygen deficiency in the HfO_2_ thin film. The oxygen vacancies generated during the HTA process might cause an additional interfacial insulating layer such as TiO_x_^41^. The large coercive voltage of HTA films observed in the P–V hysteresis measurement in Fig. 3a can be attributed to this interfacial layer. Considering the Landau-Devonshire formalism, additional insulator layers induce the reduction of spontaneous polarization value^51^ and additional RC delay in the switching dynamics causing retarded ferroelectric switching. However, even though the additional insulating interlayer can be formed, the HTA films exhibited enhanced ferroelectricity with the accelerated switching speed as shown in Fig. 4. Therefore, the enhancement of ferroelectric properties induced by the oxygen vacancies in this study might be underestimated due to the interfacial layer during the HTA process. Considering the conventional role of oxygen deficiency in ferroelectrics, it is noteworthy that the oxygen deficiency accelerated the ferroelectric switching speed without penalizing inhomogeneous switching, i.e., broadening of the Lorentzian distribution due to rigid defect dipoles. For example, the enhanced ferroelectricity during wake-up process, i.e., the increase of ferroelectric polarization during the external electric field cycling, was attributed to a reduction of defect dipoles and/or increase in ferroelectric volume fraction^46^. The annihilation of defect dipoles and/or increased ferroelectric volume fraction in ferroelectric domains resulted in homogeneous switching with slower ferroelectric characteristic switching speed. However, the increase of oxygen deficiency in ferroelectric HfO_2_ controlled by the specific thermal annealing exhibited distinctive properties with the reduced switching activation energy and faster homogenous switching.

## Conclusion

We investigated the effects of oxygen deficiency on the ferroelectric properties of Si-doped HfO_2_ films. Through the structural and chemical analyses, we confirmed increased oxygen deficiencies with negligible phase transformation during high temperature annealing. Polarization–voltage and capacitance–voltage measurements revealed enhanced ferroelectricity with increasing oxygen deficiency. The ferroelectric switching dynamics exhibited unexpectedly fast ferroelectric characteristic switching with enhanced robust ferroelectric polarization in the more oxygen-deficient Si-doped HfO_2_ film. The enhanced ferroelectricity in the oxygen deficiency-rich film was attributed to the reduced activation energy of ferroelectric dipole switching by first-principles calculations and Monte Carlo simulations.

## Methods

### Sample fabrication

HfO_2_ films doped with 4.2% Si and 8 nm thick were grown on a TiN bottom electrode by atomic layer deposition using tetrakis(dimethylamido)hafnium, tetrakis(dimethylamino)silane, and ozone. High-temperature annealing with short annealing time enabled the optimization of diverse functionality through grain size control^52,53^, active implantation of dopants with minimal diffusion^54^, and defect formation^55^. Annealing the HfO_2_ films at high temperature with short annealing time controlled the oxygen deficiency concentration (see supplementary Sect. 8). Films were post-annealed at 600 °C for 20 s (LTA) and 800 °C for 1 s (HTA) in an N_2_ atmosphere after deposition of the top Pt/TiN electrode. The Pt/TiN top electrode was patterned as a circular shape with a radius of 100 μm for electrical measurements.

### Sample characterization

The high resolution X-ray photoemission spectroscopy (XPS) measurements were conducted using monochromatic X-ray source and spherical sector analyzer (SIGMA PROBE, ThermoFisher Scientific, U.K). The core level spectra peaks were fitted using a pseudo Voigt function (a convolution of 30% Lorentzian and 70% Gaussian functions) with the Shirley type baseline subtraction. The electrical measurements were conducted after wake-up process by inducing 10,000 pulse cycles of 3 V at a frequency of 10 kHz. Polarization–voltage (P–V) curves and time-dependent dynamic polarization switching (P(t)) were conducted using a ferroelectric tester (TF Analyzer 3000; aixACCT Systems GmbH, Aachen, Germany) and a semiconductor parameter analyzer (4200-SCS; Keithley Instruments, Cleveland, OH, USA). The P–V curves were measured with the voltage pulse frequency of 2 kHz. The dielectric constant as a function of voltage was measured with the ac voltage frequency and height of 10 kHz and 100 mV. an impedance analyzer (E4990A; Agilent, Palo Alto, CA). For STEM and EELS analyses, films were fabricated into thin lamella using a focused ion beam (FEI; Helios). The lamella was observed using an aberration-corrected Titan G2 microscope (60–300 kV) operating at 200 kV.

## Supplementary Information

Supplementary Information

## References

[1] Mahajan S (1989). Growth- and processing-induced defects in semiconductors. Prog. Mater. Sci..

[2] Yazyev OV, Helm L (2007). Defect-induced magnetism in graphene. Phys. Rev. B.

[3] Schaub R (2001). Oxygen vacancies as active sites for water dissociation on rutile TiO_2_ (110). Phys. Rev. Lett..

[4] Feng Y (2020). Defects and aliovalent doping engineering in electroceramics. Chem. Rev..

[5] Lines ME, Glass AM (2001). Principles and Applications of Ferroelectrics and related Materials.

[6] Itoh M (1999). Ferroelectricity induced by oxygen isotope exchange in strontium titanate perovskite. Phys. Rev. Lett..

[7] Lee D (2012). Active control of ferroelectric switching using defect-dipole engineering. Adv. Mater..

[8] Kalinin SV (2010). Defect-mediated polarization switching in ferroelectrics and related materials: From mesoscopic mechanisms to atomistic control. Adv. Mater..

[9] Barrozo P (2020). Defect-enhanced polarization switching in the improper ferroelectric LuFeO_3_. Adv. Mater..

[10] Böscke T, Müller J, Bräuhaus D, Schröder U, Böttger U (2011). Ferroelectricity in hafnium oxide thin films. Appl. Phys. Lett..

[11] Müller J (2011). Ferroelectric Zr_0.5_Hf_0.5_O_2_ thin films for nonvolatile memory applications. Appl. Phys. Lett..

[12] Wei Y (2018). A rhombohedral ferroelectric phase in epitaxially strained Hf_0.5_Zr_0.5_O_2_ thin films. Nat. Mater..

[13] Park MH (2015). Ferroelectricity and antiferroelectricity of doped thin HfO_2_-based films. Adv. Mater..

[14] Shimizu T (2016). The demonstration of significant ferroelectricity in epitaxial Y-doped HfO_2_ film. Sci. Rep..

[15] Muller J (2012). Ferroelectricity in simple binary ZrO_2_ and HfO_2_. Nano Lett..

[16] Pešić M (2016). Physical mechanisms behind the field-cycling behavior of HfO_2_-based ferroelectric capacitors. Adv. Funct. Mater..

[17] Yurchuk, E. *et al.* HfO_2_-based Ferroelectric Field-Effect Transistors with 260 nm channel length and long data retention. In *2012 4th IEEE International Memory Workshop* 1–4 (IEEE).

[18] Müller, J. *et al.* Ferroelectric hafnium oxide: A CMOS-compatible and highly scalable approach to future ferroelectric memories. In *2013 IEEE International Electron Devices Meeting.* 10.18. 11–10.18. 14 (IEEE).

[19] Stolichnov I (2018). Genuinely ferroelectric sub-1-volt-switchable nanodomains in Hf_x_Zr_(1–x)_O_2_ ultrathin capacitors. ACS Appl. Mater. Interfaces.

[20] Sang X, Grimley ED, Schenk T, Schroeder U, LeBeau JM (2015). On the structural origins of ferroelectricity in HfO_2_ thin films. Appl. Phys. Lett..

[21] Cheema SS (2020). Enhanced ferroelectricity in ultrathin films grown directly on silicon. Nature.

[22] Böscke, T., Müller, J., Bräuhaus, D., Schröder, U. & Böttger, U. Ferroelectricity in hafnium oxide: CMOS compatible ferroelectric field effect transistors. In *2011 International Electron Devices Meeting.* 24.25. 21–24.25. 24 (IEEE).

[23] Lee K (2018). Ferroelectricity in epitaxial Y-doped HfO_2_ thin film integrated on Si substrate. Appl. Phys. Lett..

[24] Mulaosmanovic, H. *et al.* Evidence of single domain switching in hafnium oxide based FeFETs: Enabler for multi-level FeFET memory cells. In *2015 IEEE International Electron Devices Meeting (IEDM).* 26.28. 21–26.28. 23 (IEEE).

[25] Mulaosmanovic, H. *et al.* Novel ferroelectric FET based synapse for neuromorphic systems. In *2017 Symposium on VLSI Technology* T176–T177 (IEEE).

[26] Lee K (2019). Stable subloop behavior in ferroelectric Si-doped HfO_2_. ACS Appl. Mater. Interfaces.

[27] Lee H-J (2020). Scale-free ferroelectricity induced by flat phonon bands in HfO_2_. Science.

[28] Grimley ED, Schenk T, Mikolajick T, Schroeder U, LeBeau JM (2018). Atomic structure of domain and interphase boundaries in ferroelectric HfO_2_. Adv. Mater. Interfaces.

[29] Marrocchelli D, Bishop SR, Tuller HL, Yildiz B (2012). Understanding chemical expansion in non-stoichiometric oxides: Ceria and zirconia case studies. Adv. Funct. Mater..

[30] Chatzichristodoulou C, Norby P, Hendriksen PV, Mogensen MB (2015). Size of oxide vacancies in fluorite and perovskite structured oxides. J. Electroceram..

[31] Park MH (2017). A comprehensive study on the structural evolution of HfO_2_ thin films doped with various dopants. J. Mater. Chem. C.

[32] Suzer S (2003). Soft x-ray photoemission studies of Hf oxidation. J. Vac. Sci. Technol. A.

[33] Januar M (2015). The role of oxygen plasma in the formation of oxygen defects in HfO_x_ films deposited at room temperature. J. Mater. Chem. C.

[34] Kang J, Lee E-C, Chang K-J, Jin Y-G (2004). H-related defect complexes in HfO_2_: A model for positive fixed charge defects. Appl. Phys. Lett..

[35] Park MH (2013). Effect of forming gas annealing on the ferroelectric properties of Hf_0.5_Zr_0.5_O_2_ thin films with and without Pt electrodes. Appl. Phys. Lett..

[36] Hamouda W (2020). Physical chemistry of the TiN/Hf_0.5_Zr_0.5_O_2_ interface. J. Appl. Phys..

[37] Stemmer S, Chen Z, Zhu W, Ma T (2003). Electron energy-loss spectroscopy study of thin film hafnium aluminates for novel gate dielectrics. J. Microsc..

[38] Jang JH (2011). Investigation of oxygen-related defects and the electrical properties of atomic layer deposited HfO_2_ films using electron energy-loss spectroscopy. J. Appl. Phys..

[39] Wilk G, Muller D (2003). Correlation of annealing effects on local electronic structure and macroscopic electrical properties for HfO_2_ deposited by atomic layer deposition. Appl. Phys. Lett..

[40] Fengler F, Hoffmann M, Slesazeck S, Mikolajick T, Schroeder U (2018). On the relationship between field cycling and imprint in ferroelectric Hf_0.5_Zr_0.5_O_2_. J. Appl. Phys..

[41] Grimley ED (2016). Structural changes underlying field-cycling phenomena in ferroelectric HfO_2_ thin films. Adv. Electron. Mater..

[42] Cook JW, Jaffe H (1971). Applications of piezoelectric ceramics. Piezoelectric Ceram..

[43] Kohli M, Muralt P (1999). Poling of ferroelectric thin films. Ferroelectrics.

[44] Kim HJ (2016). A study on the wake-up effect of ferroelectric Hf_0.5_Zr_0.5_O_2_ films by pulse-switching measurement. Nanoscale.

[45] Jo J (2007). Domain switching kinetics in disordered ferroelectric thin films. Phys. Rev. Lett..

[46] Lee TY (2018). Ferroelectric polarization-switching dynamics and wake-up effect in Si-doped HfO_2_. ACS Appl. Mater. Interfaces.

[47] Tybell T, Paruch P, Giamarchi T, Triscone JM (2002). Domain wall creep in epitaxial ferroelectric Pb(Zr_0.2_Ti_0.8_)O_3_ thin films. Phys. Rev. Lett..

[48] Jo JY (2009). Nonlinear dynamics of domain-wall propagation in epitaxial ferroelectric thin films. Phys. Rev. Lett..

[49] Wu Y-Z, Yao D-L, Li Z-Y (2002). Monte-Carlo simulation of the switching behavior in ferroelectrics with dipolar defects. Solid State Commun..

[50] Young W, Elcock E (1966). Monte Carlo studies of vacancy migration in binary ordered alloys: I. Proc. Phys. Soc..

[51] Khan AI (2011). Experimental evidence of ferroelectric negative capacitance in nanoscale heterostructures. Appl. Phys. Lett..

[52] Hosseini S, Alishahi M, Najafizadeh A, Kermanpur A (2012). The improvement of ductility in nano/ultrafine grained low carbon steels via high temperature short time annealing. Mater. Lett..

[53] Kim M (2017). High-temperature–short-time annealing process for high-performance large-area perovskite solar cells. ACS Nano.

[54] Sedgwick T (1983). Short time annealing. J. Electrochem. Soc..

[55] Pagani M, Falster R, Fisher G, Ferrero G, Olmo M (1997). Spatial variations in oxygen precipitation in silicon after high temperature rapid thermal annealing. Appl. Phys. Lett..

