# Supplementary material for: Enhanced ferroelectric switching speed of Si-doped HfO2 thin film tailored by oxygen deficiency
Source: Sci Rep. 2021 Mar 18;11:6290. doi: 10.1038/s41598-021-85773-7 (PMC7973512; doi:10.1038/s41598-021-85773-7)
Supplement: Supplementary file 1 — Supplementary Information [file 41598_2021_85773_MOESM1_ESM.docx]

**Supporting Information**

Enhanced Ferroelectric Switching Speed of Si-doped HfO_2_ Thin Film Tailored by Oxygen Deficiency

Kyoungjun Lee^1,5^, Kunwoo Park^2,3,5^, Hyun-Jae Lee^4^, Myeong Seop Song^1^, Kyu Cheol Lee^1^, Jin Namkung^1^, Jun Hee Lee^4^, Jungwon Park^2,3^ and Seung Chul Chae^1,*^

^1^Department of Physics Education, Seoul National University, Seoul 08826, Korea

^2^School of Chemical and Biological Engineering, Institute of Chemical Process, Seoul National University, Seoul 08826, Korea

^3^Center for Nanoparticle Research, Institute for Basic Science (IBS), Seoul 08826, Korea

^4^School of Energy and Chemical Engineering, Ulsan National Institute of Science and Technology (UNIST), Ulsan 44919, Korea

^5^These authors contributed equally.

*Corresponding author

e-mail: [scchae@snu.ac.kr](mailto:scchae@snu.ac.kr)

KEYWORDS: ferroelectric, oxygen deficiency, HfO_2_, switching speed

1. Scanning transmission electron microscopy

High-resolution scanning transmission electron microscopy (HRSTEM) was used for the detailed structural analysis. Lamella for HRSTEM observation was produced by focused ion beam (FIB). The lamella was milled until the optimal thickness (20 nm) for high-resolution images was attained. The thickness was characterized using electron energy-loss spectroscopy (EELS). Precise atomic positions of films were observed in HRSTEM images for LTA and HTA films shown in Fig. S1a,b. Crystallographic structure of LTA and HTA films were characterized using Fast Fourier transformation (FFT) images corresponding to the HRSTEM images (Fig. S1c,d). Both FFT images were identical to diffraction patterns of Pca2_1_ orthorhombic phase. As shown in Fig. S1e,f, simulated atomic images based on the FFT images were similar to the HRSTEM images. HRSTEM analysis prove that both films were mostly composed of Pca2_1_ orthorhombic phase with negligible other structure, such as monoclinic and tetragonal phases.

2. Grain size estimation

The grain size of LTA and HTA films was estimated by high resolution transmission electron microscope (HRTEM) images. Fig. S2 shows the observed C_s_ corrected atomic resolution HRTEM images of LTA and HTA films. Through the utilizing the geometric phase analysis (GPA) the strain maps were obtained. The strain maps as shown in Fig. S2 display grain boundaries between domain consisting of HfO_2_ films as well as the interfaces between HfO_2_ film and TiN electrodes. For the accurate estimation of grain size, the HRTEM images were repeatedly obtained along the entire lamella specimen with atomic resolution. Based on the obtained strain maps from atomic resolution HRTEM images and their GPA analysis, the averaged projected grain size was estimated about 240 nm^2^. The size of grains comprising LTA and HTA films was almost identical. Considering the thickness of films (~ 8 nm), the width of the grains can be estimated as ~ 30 nm.

3. Electron energy-loss spectroscopy analyses

We used EELS to probe the electronic structure of the Si-doped HfO_2_ thin films. Oxygen *K* edge and valence EELS (VEELS) spectra were collected simultaneously in the STEM mode with dual-range EELS (Fig. S3). The VEELS spectrum in Fig. S3a features peaks marked as A–F. Peak A corresponds to the single-electron transition between the O 2p and Hf 5d states, and peak B corresponds to plasmon excitation.^1^ Energy-loss peaks above 30 eV (peaks C–F) were due to HfO_2_ edges, which are related to the bond angles, bond lengths, and coordination.^1-3^ Few differences were observed in the intensities of peaks C–F between the LTA and HTA film; this was interpreted as similar crystallinity and corresponded to the results shown in Fig. 1a,b and Fig 2a,b. However, the A:B intensity ratio B differed between the LTA and HTA films. Because peak A originates from the interband transition and peak B corresponds to plasmon loss, point defects such as oxygen vacancies could lower the intensity of peak A and not significantly affect the intensity of peak B. Hence, the oxygen vacancy concentration was determined using the A:B peak intensity ratio (Fig. S3). Features at the O *K* edges also confirmed the difference in oxygen vacancy concentration between the LTA and HTA films. The G, H pair of peaks in Fig. S3b was due to hybridization of the O 2*p* component with the Hf *d*–e_g_ and *d*–t_2g_ components.^4^ Small peaks above 540 eV were due to hybridization between the O 2*p* and Hf *s,p* components. Because the doublet was due to the overlap between O and Hf orbitals, oxygen vacancies could weaken this feature. The doublet feature was diminished in the HTA film because peak G was broader and the energy gap between peaks G and H was smaller compared to the LTA film. Therefore, EELS analyses confirmed that the concentration of oxygen vacancies was higher in the HTA film than in the LTA film.

4. Current-voltage curves for the pinched hysteresis

To clarify the pinched hysteresis due to the defect dipoles in Fig. 3a, the current-voltage curves were shown in Fig. S4. The peak splitting behavior of current-voltage curves can be attributed to the internal bias field due to the vacancy-rich interfacial layers.^5^ Even though we measured the current-voltage curves after wake-up process, the internal bias field related to the defect dipoles did not fully disappear. This can be attributed to the defect-dipole rich bottom interface where pinned domains were stabilized during the thermal annealing process.^6^ These current-voltages characteristics can be considered as clear evidence of internal bias field due to the defect dipoles.

5. Details of switching dynamics

The ferroelectric switching dynamics in terms of the time and voltage were measured through the switched polarization value ΔP after applying the voltage pulses as follow. For the negatively poled polarization state, a triangular pulse of −3.5 V at 2 kHz was applied to the top electrode of pristine sample. To switch polarization, square-shaped voltage pulses were applied subsequently to the top electrode over the range of 0.5-3 V and width of 100 ns to 1 ms. To read the switched polarization value ΔP, we applied triangular pulse of -3.5 V at 2 kHz twice. The former pulse consists of the switching current and non-switching current, while the latter pulse consists of only non-switching current. The spontaneous polarization value 2P_s_ was measured by applying the reading pulse twice at the negatively polled specimen. Considering the poly crystalline nature of films, the measured switching dynamics were fitted through the nucleation-limited switching (NLS) model considering the Lorentzian distribution for the characteristic switching time as below.

$\frac{\Delta P\left( t \right)}{2P_{s}}=\int_{-\infty}^{\infty} \left[ 1-exp\left\{ -\left( \frac{t}{t_{0}} \right)^{2} \right\} \right]F\left( \log t_{0} \right)d(\log t_{0})$

where

$F\left( \log t_{0} \right)=\frac{A}{\pi}\left[ \frac{w}{\left( \log t_{0}-\log t_{1} \right)^{2}+w^{2}} \right]$

*A, w,* and *log t_1_* indicate a normalization constant, the half width at half-maximum of the distribution, and the mean value of the distribution, respectively.

6. Theoretical calculations

First-principle calculations based on density functional theory were conducted using the Vienna Ab initio simulation package code (VASP Software GmBH, Vienna, Austria) to estimate the energy barrier during ferroelectric dipole flipping for different oxygen vacancy concentrations.^7-10^ We used the generalized gradient approximation method to describe the exchange–correlation functional and pseudopotentials generated under the projector-augmented plane wave scheme of Perdew, Burke, and Ernzerhof.^11,12^ The Monkhorst–Pack method involving integration within the Brillouin zone was used with a 4 × 4 × 4 k-point grid.^13^ The energy cut-off for the plane-wave basis was set to 500 eV, and the force criterion for structure optimization was 0.01 eV/Å. The stabilized unit cells contained four formula units: 0% oxygen vacancy concentration for orthorhombic HfO_2_ with a = 5.2733 Å, b = 5.0551 Å, and c = 5.0851 Å. To optimize structures with oxygen vacancies, we used 2×2×2 supercells containing 2×2×2 k-point grid with 32 formula units. Actually, there are a lot of possible configurations for oxygen vacancies. Because of computational cost issue, among a hundred of randomly generated structure with oxygen vacancies, we used the most stable structure with a = 10.5160 Å, b= 10.0942 Å, c = 10.13751 Å, α = 90.1073°, β = 90.0470°, and γ = 89.7772°. The Berry phase method was used to calculate the electric spontaneous polarization.^14,15^ The calculated spontaneous polarizations for 0% and 6.25% oxygen vacancy concentrations were 41.59 and 42.52 μC/cm^2^, respectively. The remnant polarization increased with increasing oxygen vacancy concentration, which was in agreement with the experimental results (Fig. 3).

7. Details of Monte Carlo simulations

The effect of activation energy on the ferroelectric switching was investigated through the Monte Carlo simulations considering the long-range dipole-dipole interaction. The simulations were performed under the conditions considering the previous studies.^16-19^ The square 100 × 100 lattices were used with periodic boundary conditions to prevent the rare events such as back switching and time autocorrelation due to the finite size effect. During the simulations, we used the values as follows: *kT* = 1 (arb. units) and an external field *E* = 12 (arb. units). The dipole-dipole interactions were considered up to the third neighborhood for the converged mean switching time.

8. Effects of the low temperature annealing (LTA) process with different times

To confirm the annealing time dependence of ferroelectric HfO_2_, we conducted LTA process with different times. Figure S5 shows the P-V and C-V curves of LTA films with different times. As the annealing time increased, the remnant polarization value decreased. Also, the dielectric constant at the high voltage was reduced as the annealing time increased. Considering the dielectric constants of monoclinic (~16), orthorhombic (~27), and tetragonal (~28) HfO_2_ phases,^20^ the increment of the monoclinic phase portion can be expected from the decrease of the dielectric constant after the thermal annealing process.^21^ Also, the LTA films annealed during 2,000 seconds exhibited nearly hysteresis-free C-V curves indicating the reduction of relative ferroelectric orthorhombic phase. The increment of annealing time can expand the grain size of the HfO_2_ phase. Considering the energetically stable monoclinic phase preferred in the bulk HfO_2_, the fraction of the monoclinic phase can increase in the enlarged grains of HfO_2_ film.^22^ The above annealing time dependence of C-V curves clarifies the reason why we used HTA with short time methods.


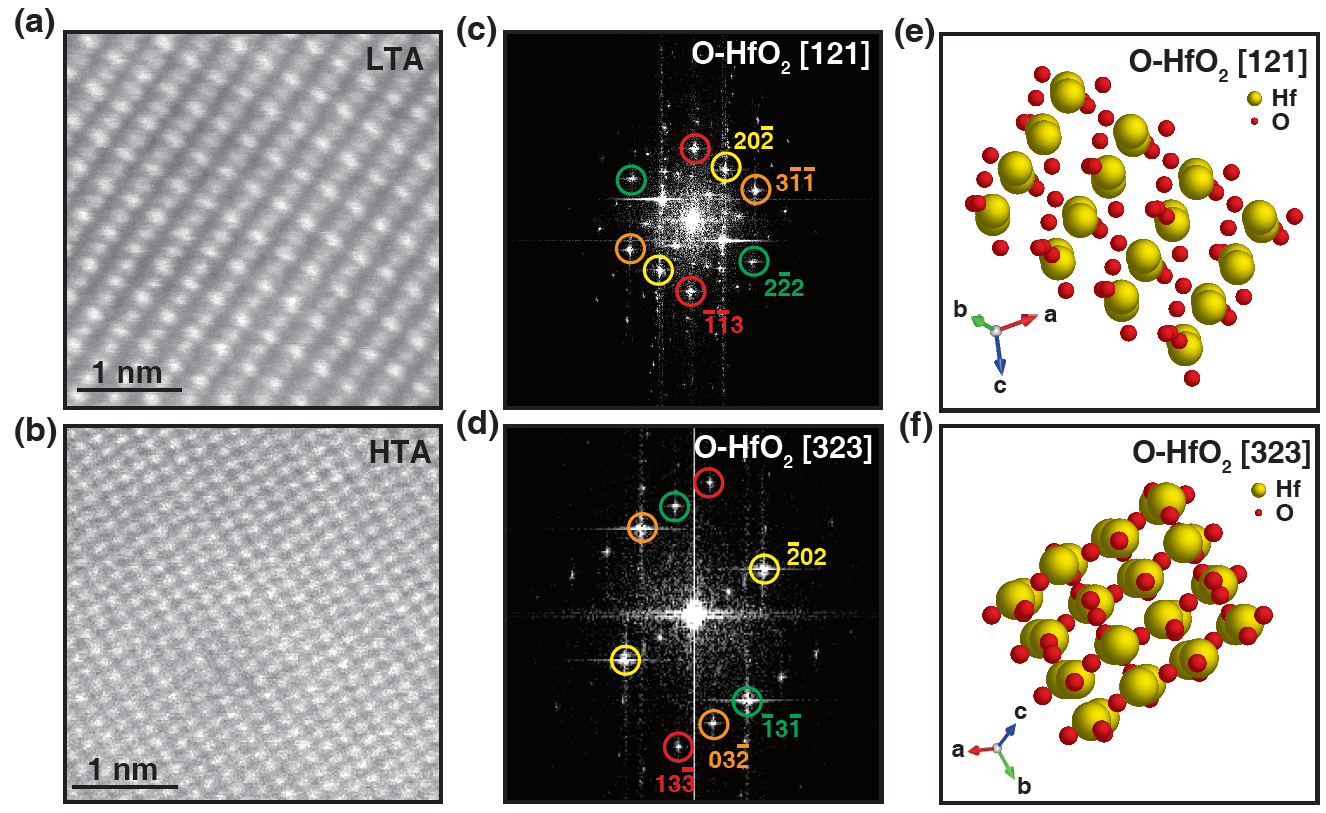


Fig. S1 High-resolution scanning transmission electron microscope cross-sectional images of the (a) LTA and (b) HTA films. Corresponding fast Fourier transform (FFT) patterns of the (c) LTA and (d) HTA films. Simulated atomic images based on FFT images of the (e) LTA and (f) HTA films.


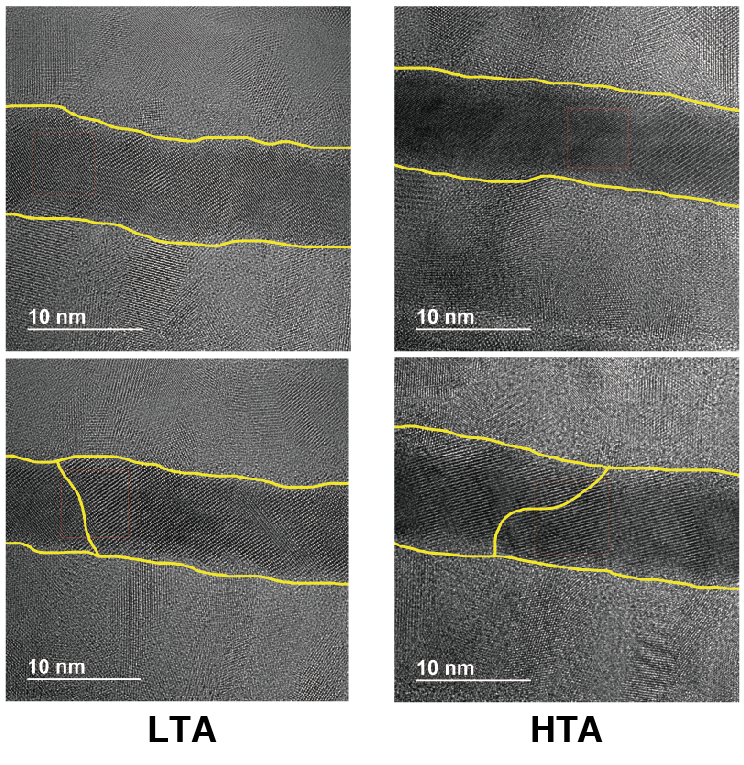


Fig. S2 A cross sectional HRTEM images of LTA and HTA films with indication of grain boundaries (yellow lines).

Fig. S3 Electron energy-loss spectra. (a) Valence electron energy-loss spectrum and (b) O K-edge of the Si-doped HfO_2_ LTA thin film.

Fig. S4 Current-Voltage curves of LTA films

Fig. S5 (a)Polarization-Voltage and (b)Capacitance-Voltage curves of LTA films with different annealing times.

**References**

1 Agustin, M. P., Fonseca, L. R., Hooker, J. C. & Stemmer, S. Scanning transmission electron microscopy of gate stacks with HfO_2_ dielectrics and TiN electrodes. *Appl. Phys. Lett.* **87**, 121909 (2005).

2 Stemmer, S., Chen, Z., Zhu, W. & Ma, T. Electron energy‐loss spectroscopy study of thin film hafnium aluminates for novel gate dielectrics. *J. Microsc.* **210**, 74-79 (2003).

3 Jang, J. H. *et al.* Investigation of oxygen-related defects and the electrical properties of atomic layer deposited HfO_2_ films using electron energy-loss spectroscopy. *J. Appl. Phys.* **109**, 023718 (2011).

4 Mizoguchi, T., Saitoh, M. & Ikuhara, Y. First-principles calculation of oxygen K-electron energy loss near edge structure of HfO_2_. *J. Phys. Condens. Matter* **21**, 104212 (2009).

5 Richter, C. *et al.* Si Doped Hafnium Oxide—A “Fragile” Ferroelectric System. *Adv. Electron. Mater.* **3**, 1700131 (2017).

6 Lee, D. *et al.* Active control of ferroelectric switching using defect‐dipole engineering. *Adv. Mater.* **24**, 6490-6495 (2012).

7 Kresse, G. & Hafner, J. Ab initio molecular dynamics for liquid metals. *Phys. Rev. B* **47**, 558 (1993).

8 Kresse, G. & Furthmüller, J. Efficient iterative schemes for ab initio total-energy calculations using a plane-wave basis set. *Phys. Rev. B* **54**, 11169 (1996).

9 Kresse, G. & Furthmüller, J. Efficiency of ab-initio total energy calculations for metals and semiconductors using a plane-wave basis set. *Comput. Mater. Sci.* **6**, 15-50 (1996).

10 Kresse, G. & Joubert, D. From ultrasoft pseudopotentials to the projector augmented-wave method. *Phys. Rev. B* **59**, 1758 (1999).

11 Blöchl, P. E. Projector augmented-wave method. *Phys. Rev. B* **50**, 17953 (1994).

12 Perdew, J. P., Burke, K. & Ernzerhof, M. Generalized gradient approximation made simple. *Phys. Rev. Lett.* **77**, 3865 (1996).

13 Monkhorst, H. J. & Pack, J. D. Special points for Brillouin-zone integrations. *Phys. Rev. B* **13**, 5188 (1976).

14 King-Smith, R. & Vanderbilt, D. Theory of polarization of crystalline solids. *Phys. Rev. B* **47**, 1651 (1993).

15 Vanderbilt, D. & King-Smith, R. Electric polarization as a bulk quantity and its relation to surface charge. *Phys. Rev. B* **48**, 4442 (1993).

16 Wu, Y.-Z., Yao, D.-L. & Li, Z.-Y. Monte-Carlo simulation of the switching behavior in ferroelectrics with dipolar defects. *Solid State Commun.* **122**, 395-400 (2002).

17 Kühn, M. & Kliem, H. Monte Carlo simulations of ferroelectric properties based on a microscopic model for PVDF. *Phys. Status Solid B* **245**, 213-223 (2008).

18 Lee, T. Y. *et al.* Ferroelectric polarization-switching dynamics and wake-up effect in Si-doped HfO_2_. *ACS Appl. Mater. Interfaces* **11**, 3142-3149 (2018).

19 Lee, K. *et al.* Stable subloop behavior in ferroelectric Si-doped HfO_2_. *ACS Appl. Mater. Interfaces* **11**, 38929-38936 (2019).

20 Materlik, R., Künneth, C. & Kersch, A. The origin of ferroelectricity in Hf_1−x_Zr_x_O_2_: A computational investigation and a surface energy model. *J. Appl. Phys.* **117**, 134109 (2015).

21 Grimley, E. D. *et al.* Structural changes underlying field‐cycling phenomena in ferroelectric HfO_2_ thin films. *Adv. Electron. Mater.* **2**, 1600173 (2016).

22 Lee, Y. H. *et al.* Nucleation-limited ferroelectric orthorhombic phase formation in Hf_0.5_Zr_0.5_O_2_ thin films. *Adv. Electron. Mater.* **5**, 1800436 (2019).
